# Speed of Evolution and Correlations in Multi-Mode Bosonic Systems

**DOI:** 10.3390/e24121774

**Published:** 2022-12-05

**Authors:** Alexei D. Kiselev, Ali Ranim, Andrei V. Rybin

**Affiliations:** 1 Laboratory of Quantum Processes and Measurements, ITMO University, Kadetskaya Line 3b, 199034 Saint Petersburg, Russia; 2 School of Physics and Engineering, ITMO University, Kronverksky Pr. 49, bldg. A, 197101 Saint Petersburg, Russia; 3 Center of Information Optical Technology, ITMO University, Birzhevaya Line 14a, 199034 Saint Petersburg, Russia

**Keywords:** quantum speed limits, open continuous variable quantum systems, intermode couplings, squeezed states, disentanglement, mutual information

## Abstract

We employ an exact solution of the thermal bath Lindblad master equation with the Liouvillian superoperator that takes into account both dynamic and environment-induced intermode couplings to study the speed of evolution and quantum speed limit (QSL) times of a open multi-mode bosonic system. The time-dependent QSL times are defined from quantum speed limits, giving upper bounds on the rate of change of two different measures of distinguishability: the fidelity of evolution and the Hilbert–Schmidt distance. For Gaussian states, we derive explicit expressions for the evolution speed and the QSL times. General analytical results are applied to the special case of a two-mode system where the intermode couplings can be characterized by two intermode coupling vectors: the frequency vector and the relaxation rate vector. For the system initially prepared in a two-mode squeezed state, dynamical regimes are generally determined by the intermode coupling vectors, the squeezing parameter and temperature. When the vectors are parallel, different regimes may be associated with the disentanglement time, which is found to be an increasing (a decreasing) function of the length of the relaxation vector when the squeezing parameter is below (above) its temperature-dependent critical value. Alternatively, we study dynamical regimes related to the long-time asymptotic behavior of the QSL times, which is characterized by linear time dependence with the proportionality coefficients defined as the long-time asymptotic ratios. These coefficients are evaluated as a function of the squeezing parameter at varying temperatures and relaxation vector lengths. We also discuss how the magnitude and orientation of the intermode coupling vectors influence the maximum speed of evolution and dynamics of the entropy and the mutual information.

## 1. Introduction

Among a wide range of multifaceted fundamental problems dealing with different aspects of dynamics of quantum systems, the importance of the problems related to the speed of evolution of a quantum state for quantum technologies such as quantum communications and quantum computations cannot be overestimated. In processes underlying these technologies, this speed generally determines how fast a given task can be processed.

The maximum speed of the evolution can be quantified using the quantum speed limit (QSL) time defined as the minimal time needed for a system to perform transition between predetermined initial and final (target) states. An important point is that quantum mechanics dictates QSL bounds for the minimal evolution time which is inversely related to the evolution speed. For the case of unitary evolution, the well-known Mandelstam–Tamm [1,2] and Margolus–Levitin [3,4,5] inequalities provide general limits on the speed of dynamical evolution and set a dynamical intrinsic time scale. Interesting alternative geometric derivations for both inequalities are obtained from the statistical distance between quantum states in [6].

In [7], the geometric approach was used to generalize the inequalities to the case of nonunitary dynamics of open quantum systems. Following a similar general approach, where quantum speed limits are derived as upper bounds on the rate of change of a geometric measure of distinguishability, different expressions for generalized QSL bounds were also obtained in [8,9] using quantum Fisher information and the relative purity. The QSL inequality for open quantum systems using the trace norm was derived and interpreted in [10]. More details on QSLs and their applications in the fields such as quantum metrology and optimal control theory can be found in, e.g., Ref. [11] (see also references cited therein).

Recently, in Ref. [12], the QSL approach was applied to solve the optimal control problem dealing with the minimum energetic cost required to implement a quantum gate. Speed limits on the informational measures such as the von Neumann entropy, maximal information, and coherence of quantum systems were introduced in [13] where the speed limit on the maximal information is used to obtain the minimum time required to erase the information of quantum systems via some quantum processes of interest.

Quantum navigation is another important class of controlled quantum dynamics whereby the objective is to transport one quantum state into another in the shortest possible time under the influence of uncontrollable perturbations. Problems of this kind can be regarded as the quantum counterpart of the classical Zermelo navigation problem of finding the time-optimal control that takes a ship from one location to another, under the influence of external wind or currents [14,15,16,17]. Analysis of the evolution speed is a necessary step toward solving the quantum Zermelo problem.

For the last half of the decade, the evolution speed of two-level open quantum systems interacting with both Markovian and non-Markovian environments has been the subject of intense studies [16,18,19,20,21]. A systematic analysis of the most common QSL bounds in the damped Jaynes–Cummings model was performed in [22]. The problem of quantum metrology in the context of non-Markovian quantum evolution of a two-qubit system was explored in [23].

As compared to the case of finite dimensional quantum systems, for bosonic multi-mode quantum systems representing a family of open continuous-variable systems, the problem of evolution speed has not yet received a proper attention. In this paper, we intend to fill the gap.

In a recent paper [24], the evolution speed and various QSL times were computed for the special case of a single quantized cavity mode that undergoes Markovian dynamics. Obviously, in order to study the effects determined by dynamics of intermode correlations, we shall need to go beyond the scope of single-mode models. These effects are governed by the intermode interactions arising from both dynamic and environment mediated couplings.

For instance, when a two-mode boson system represents the polarization degrees of freedom of of a quantized radiation mode [25], these couplings may have a profound effect on dynamics of the averages of the quantum Stokes operators that underlies the depolarization of quantum light propagating in a fiber [26,27,28]. Note that a similar two-boson system was formulated in Ref. [29] to model two cavity modes interacting with a single three-level atom.

As far as correlations are concerned, there is an extensive body of the literature dealing with dynamics of entanglement in open continuous-variable systems such as two coupled oscillators [30,31,32,33,34,35,36,37,38,39,40,41,42,43] (see also Refs. [44,45] for reviews). It turned out that the decay time of entanglement (the time of disentanglement) may be shorter than the time of decoherence and, under certain conditions, finite-time disentanglement commonly known as the “sudden death of entanglement” [46,47,48,49,50,51] may also take place in continuous-variable systems [33,39,43].

In this paper, we explore how the evolution speed of multi-mode bosonic systems is influenced by the dynamical regimes dictated by the intermode couplings. The plan of this paper is as follows.

Section 2 begins with a brief discussion of QSL. We will introduce the fidelity of evolution and the Hilbert–Schmidt (HS) distance as measures of the distinguishability of quantum states applicable to the special case of a pure initial state [24]. For the rates of change of both of these measures, the speed of evolution appears as the upper bound that defines the time-dependent QSL times. In Section 2.1, our starting point is the Gorini–Kossakowski–Sudarshan–Lindblad (GKSL) master equation (the general form of quantum kinetic equations which preserves the complete positivity of the dynamics [52,53,54]) for the multi-mode bosonic system interacting with the thermal bath and we, following Refs. [43,55], present its exact solution derived by solving the dynamical equation for the normally ordered characteristic function. In Section 2.2, we deduce general formulas that characterize the temporal evolution of Gaussian states using the generalized two-point fidelity, the evolution speed and the covariance matrix. In Section 3, general theoretical results are applied to the special case of a two-mode bosonic system which can be regarded as a model describing the propagation of quantized polarization modes in an optical fiber [27]. For this system, the intermode interactions can be conveniently treated using a geometric representation based on the frequency and the relaxation rate vectors.

In Section 4, we present a number of closed-form analytical relations for the cases where these vectors are either parallel or mutually orthogonal. For the case where the system is initially prepared in the two-mode squeezed state, different dynamical regimes are studied depending on the squeezing parameter and temperature. Analytical results are used to compute the disentanglement time and dynamics of the purity, the fidelity and the HS distance. Squeezing the parameter dependence of the coefficients characterizing long-time asymptotics of the QSL times is evaluated at different temperatures and relaxation rates. We also study how the orientation of the frequency and the relaxation rate vectors influence the maximum speed of evolution and dynamics of the entropy and the mutual information.

Finally, in Section 5, we draw the results together and make some concluding remarks. Technical details on algebraic identities related to the Liouvillian superoperator and its conjugate are relegated to Appendix A. In Appendix B, a closed-form formula for the normally ordered characteristic function is used to obtain the expression for the kernel of the Green’s function describing the evolution of the Glauber–Sudarshan *P* function.

## 2. General Approach

We consider an open multi-mode bosonic system initially prepared in a pure quantum state $\hat{\rho }\left(0\right)=|\psi_{0}\rangle \langle \psi_{0}|$ and then left to evolve under Markovian dynamics. One of the widely used measures of distinguishability between the initial state $\hat{\rho }\left(0\right)$ and the evolved one $\hat{\rho }\left(t\right)$ is the trace distance, $∥\hat{\rho }\left(t\right)-\hat{\rho }{\left(0\right)∥}_{1}=\mathrm{Tr}\left|\hat{\rho }\left(t\right)-\hat{\rho }\left(0\right)\right|$, where $|\hat{A}|=\sqrt{{\hat{A}}^{†}\hat{A}}$ and the dagger denotes Hermitian conjugation.

This distance satisfy the inequalities (see, e.g., the book [56])

(1) $$\begin{array}{cc} \; & 2-2\sqrt{F_{U}\left(\hat{\rho }\left(0\right),\hat{\rho }\left(t\right)\right)}\leq \left|\right|\hat{\rho }\left(t\right)-\hat{\rho }\left(0\right){\left|\right|}_{1}\\ \; & \leq 2\sqrt{1-F_{U}\left(\hat{\rho }\left(0\right),\hat{\rho }\left(t\right)\right)} \end{array}$$

that involve the Uhlmann fidelity $F_{U}\left(\hat{\rho }\left(0\right),\hat{\rho }\left(t\right)\right)=\left|\right|\sqrt{\hat{\rho }\left(0\right)}\sqrt{\hat{\rho }\left(t\right)}{\left|\right|}_{1}^{2}$. Clearly, the inequalities (1) can also be regarded as relations linking the temporal evolution of the trace distance and dynamics of the Uhlmann fidelity that defines the geometric characteristics of density matrix operators such as the Bures distance and the Bures angle.

An important point is that in our case, the initial state $\hat{\rho }\left(0\right)$ is pure state and the Uhlmann fidelity can be simplified to the form:

(2) $$\begin{array}{cc} \; & F_{U}\left(\hat{\rho }\left(0\right),\hat{\rho }\left(t\right)\right)=\langle \psi_{0}|\hat{\rho }\left(t\right)|\psi_{0}\rangle \\ \; & =F\left(t\right)\equiv \mathrm{Tr}[\hat{\rho }\left(0\right)\hat{\rho }\left(t\right)] \end{array}$$

which is identical to the so-called *fidelity of evolution*, F(t). A two-point generalization of this fidelity

(3) $$\begin{array}{c} F\left(t_{1},t_{2}\right)\equiv \mathrm{Tr}\left[\hat{\rho }\left(t_{1}\right)\hat{\rho }\left(t_{2}\right)\right] \end{array}$$

reduces to the *purity* and the fidelity of evolution

(4) $$\begin{array}{c} F\left(t,t\right)=\mathrm{Tr}[{\hat{\rho }}^{2}\left(t\right)]=P\left(t\right),\;F\left(0,t\right)=F\left(t\right) \end{array}$$

in the limiting cases with $t_{1}=t_{2}=t$ and $t_{1}=0$, $t_{2}=t$, respectively. It will also be used to compute the *speed of evolution*:

(5) $$\begin{array}{c} v\left(t\right)\equiv {∥\frac{\partial \hat{\rho }\left(t\right)}{\partial t}∥}_{2}=\sqrt{\mathrm{Tr}{\left[\frac{\partial \hat{\rho }\left(t\right)}{\partial t}\right]}^{2}}. \end{array}$$

This speed determines the upper bound for the rate of change of the fidelity (2)

(6) $$\begin{array}{c} |\dot{F}\left(0,t\right)|\leq v\left(t\right), \end{array}$$

where a dot stands for the derivative with respect to time, which is an immediate consequence of the well-known Cauchy–Schwarz inequality for the inner product of Hilbert–Schmidt operators:

(7) $$\begin{array}{cc} \; & |\mathrm{Tr}{\hat{A}}^{†}\hat{B}|\leq ∥\hat{A}{∥}_{2}{∥\hat{B}∥}_{2} \end{array}$$

where $∥\hat{A}{∥}_{2}=\sqrt{\mathrm{Tr}|\hat{A}{|}^{2}}$ is the Hilbert–Schmidt norm.

Interestingly, the *Hilbert–Schmidt (HS) distance*, $∥\hat{\rho }\left(t\right)-\hat{\rho }{\left(0\right)∥}_{2}=\sqrt{1-2F(t)+P(t)}$, can also be used as a measure of distinguishability, and it is not difficult to show that the speed of evolution (5) provides the upper bound for the magnitude of its rate of change

(8) $$\begin{array}{cc} \; & \left|\frac{\partial }{\partial t}{∥\hat{\rho }\left(t\right)-\hat{\rho }\left(0\right)∥}_{2}\right|\leq v\left(t\right) \end{array}$$

Since $F\left(t\right)\leq \sqrt{P(t)}$ and $1-F\left(t\right)\leq ∥\hat{\rho }\left(t\right)-\hat{\rho }{\left(0\right)∥}_{2}$, we have the time-dependent *quantum speed limit (QSL) times*

(9) $$\begin{array}{cc} \; & t_{HS}\left(t\right)=\frac{∥\hat{\rho }\left(t\right)-\hat{\rho }{\left(0\right)∥}_{2}}{{\langle v\rangle }_{t}}, \end{array}$$

(10) $$\begin{array}{cc} \; & t_{F}\left(t\right)=\frac{1-F(t)}{{\langle v\rangle }_{t}},\;{\langle v\rangle }_{t}\equiv \frac{1}{t}\int_{0}^{t}v\left(\tau \right)d\tau \end{array}$$

which are associated with Equations (8) and (6) and lead to the QSL inequalities:

(11) $$\begin{array}{c} t\geq t_{HS}\geq t_{F}. \end{array}$$

From Equation (11), it might be concluded that the quantum speed limit imposed by $t_{HS}$ is tighter than the fidelity-based one (see Ref. [24] for discussion). Clearly, all the above QSL times given by Equations (9) and (10) depend on the speed and the fidelity of evolution (see Equations (5) and (2)) along with the purity (see Equation (4)).

In the remaining part of this section, our task is to provide general analytical expressions for these dynamical characteristics. More specifically, in Section 2.1, we, following our previous studies [43,55], present an exactly solvable model of Lindblad dynamics and its solution in the form suitable for our purposes. Then, in Section 2.2, we apply the results to deduce general relations for Gaussian states.

### 2.1. Master Equation and Characteristic Functions

The general form of the master equation

(12) $$\begin{array}{c} \frac{\partial \hat{\rho }}{\partial t}=L\hat{\rho } \end{array}$$

suggests that the temporal evolution of the density matrix $\hat{\rho }$ representing the state of an open quantum system is governed by the *Liouvillian superoperator*, L. In this paper, we consider the thermal bath version of the Liouvillian superoperator describing the Lindblad dynamics of an *N*-mode bosonic system in the Markovian approximation. This superoperator

(13) $$\begin{array}{cc} \; & L=-i\sum_{n,m=1}^{N}\Omega_{nm}C_{{\hat{a}}_{n}^{†}{\hat{a}}_{m}}\\ \; & +\sum_{n,m=1}^{N}K_{nm}\left(D_{{\hat{a}}_{m}{\hat{a}}_{n}^{†}}+e^{-z_{T}}D_{{\hat{a}}_{n}^{†}{\hat{a}}_{m}}\right) \end{array}$$

is expressed in terms of two superoperators: the commutator $C_{\hat{A}\hat{B}}$ given by

(14) $$\begin{array}{cc} \; & C_{\hat{A}\hat{B}}:\hat{\rho }\mapsto C_{\hat{A}\hat{B}}\hat{\rho }=\left[\hat{A}\hat{B},\hat{\rho }\right] \end{array}$$

and the the dissipator $D_{\hat{A}\hat{B}}$ given by

(15) $$\begin{array}{cc} \; & D_{\hat{A}\hat{B}}:\hat{\rho }\mapsto D_{\hat{A}\hat{B}}\hat{\rho }=2\hat{A}\hat{\rho }\hat{B}-\left\{\hat{B}\hat{A},\hat{\rho }\right\}, \end{array}$$

where the dagger denotes Hermitian conjugation; ${\hat{a}}_{n}^{†}$ (${\hat{a}}_{n}$) is the creation (annihilation) operator of the *n*th mode; $\left[\hat{A},\hat{B}\right]=\hat{A}\hat{B}-\hat{B}\hat{A}$ and $\left\{\hat{A},\hat{B}\right\}=\hat{A}\hat{B}+\hat{B}\hat{A}$ stand for the commutator and the anticommutator, respectively; $\Omega_{nm}$ ($K_{nm}$) is the element of the *frequency (dissipation) matrix*, Ω (K); $z_{T}$=$\frac{\hbar \Omega_{0}}{k_{B}T}$ is the dimensionless *inverse temperature parameter*, where $\Omega_{0}$ is the bare frequency, *ℏ* is the reduced Planck constant, $k_{B}$ is the Boltzmann constant and *T* is the temperature of the environment. The frequency and dissipation matrices are both Hermitian: $\Omega =\Omega^{†}$ and $K=K^{†}$. The dissipation matrix is also positive definite: K>0.

It is well known that averages of the displacement operators, $\hat{D}\left(\alpha \right)$ and ${\hat{D}}_{N}\left(\alpha \right)$, given by

(16) $$\begin{array}{cc} \; & \hat{D}\left(\alpha \right)=e^{\left(\alpha ,{\hat{a}}^{†}\right)-\left(\alpha^{*},\hat{a}\right)}=e^{-{\left|\alpha \right|}^{2}/2}{\hat{D}}_{N}\left(\alpha \right), \end{array}$$

(17) $$\begin{array}{cc} \; & {\hat{D}}_{N}\left(\alpha \right)\equiv :\hat{D}\left(\alpha \right):=e^{\left(\alpha ,{\hat{a}}^{†}\right)}e^{-(\alpha^{*},\hat{a})} \end{array}$$

where

(18) $$\begin{array}{cc} \; & \left(\alpha^{*},\hat{a}\right)\equiv \sum_{i=1}^{N}\alpha_{i}^{*}{\hat{a}}_{i},\;\left(\alpha ,{\hat{a}}^{†}\right)\equiv \sum_{i=1}^{N}\alpha_{i}{\hat{a}}_{i}^{†}, \end{array}$$

$f\left(\alpha \right)\equiv f\left(\alpha_{1},\cdots ,\alpha_{N},\alpha_{1}^{*},\cdots ,\alpha_{N}^{*}\right)$ and an asterisk will indicate complex conjugation, define the *symmetrically and normally ordered characteristic functions*, χ(α) and $\chi_{N}\left(\alpha \right)$, as follows

(19) $$\begin{array}{cc} \; & \chi \left(\alpha \right)=\langle \hat{D}\left(\alpha \right)\rangle =e^{-{\left|\alpha \right|}^{2}/2}\chi_{N}\left(\alpha \right),\\ \; & \chi_{N}\left(\alpha \right)=\langle {\hat{D}}_{N}\left(\alpha \right)\rangle , \end{array}$$

where $\langle \hat{O}\rangle =\mathrm{Tr}\left\{\hat{O}\hat{\rho }\right\}$.

The well-known Fourier–Weyl formula (see, e.g., the books [57,58])

(20) $$\begin{array}{cc} \; & \hat{\rho }=\int d\mu \left(\alpha \right)\mathrm{Tr}\left\{\hat{D}\left(\alpha \right)\hat{\rho }\right\}\hat{D}\left(-\alpha \right) \end{array}$$

(21) $$\begin{array}{cc} \; & d\mu \left(\alpha \right)=\prod_{i=1}^{N}d\mu \left(\alpha_{i}\right),\;d\mu \left(\alpha_{i}\right)=\frac{d^{2}\alpha_{i}}{\pi }, \end{array}$$

in combination with the orthogonality relations

(22) $$\begin{array}{c} \mathrm{Tr}\left\{\hat{D}\left(\alpha \right)\hat{D}\left(\beta \right)\right\}=\pi^{N}\delta \left(\alpha +\beta \right) \end{array}$$

can be used to obtain the identity

(23) $$\begin{array}{cc} \; & \mathrm{Tr}\left\{{\hat{\rho }}_{1}{\hat{\rho }}_{2}\right\}=\int d\mu \left(\alpha \right)\chi_{1}\left(-\alpha \right)\chi_{2}\left(\alpha \right)\\ \; & =\int d\mu \left(\alpha \right)\chi_{1}^{*}\left(\alpha \right)\chi_{2}\left(\alpha \right) \end{array}$$

linking the HS scalar product of the density operators, ${\hat{\rho }}_{1}$ and ${\hat{\rho }}_{2}$, and the inner product of their characteristic functions, $\chi_{1}\left(\alpha \right)$ and $\chi_{2}\left(\alpha \right)$. In what follows, this identity will be employed to evaluate the purity and the evolution fidelity needed to characterize the speed of evolution.

The dynamics of the normally ordered characteristic function, $\chi_{N}$, is governed by the evolution equation

(24) $$\begin{array}{cc} \; & {\dot{\chi }}_{N}\left(\alpha ,t\right)=\mathrm{Tr}\left\{\hat{D_{N}}\left(\alpha \right)\frac{\partial \hat{\rho }}{\partial t}\right\}=\mathrm{Tr}\left\{{\hat{D}}_{N}\left(\alpha \right)L\hat{\rho }\right\}\\ \; & =\mathrm{Tr}\left\{L^{†}\left({\hat{D}}_{N}\left(\alpha \right)\right)\hat{\rho }\right\}={\hat{L}}^{†}\chi_{N}\left(\alpha ,t\right) \end{array}$$

with the differential operator, ${\hat{L}}^{†}$, determined by action of the conjugate of the Liouvillian superoperator, $L^{†}$, on the displacement operator, $:\hat{D}:$ (see Appendix A for details). This operator is given by

(25) $$\begin{array}{cc} \; & {\hat{L}}^{†}=i\sum_{n,m=1}^{N}\Omega_{nm}{\hat{D}}_{mn}^{\left(-\right)}\\ \; & -\sum_{n,m=1}^{N}\Gamma_{nm}\left({\hat{D}}_{mn}^{\left(+\right)}+2n_{T}\alpha_{m}\alpha_{n}^{*}\right), \end{array}$$

where

(26) $$\begin{array}{c} {\hat{D}}_{mn}^{\left(\pm \right)}=\alpha_{m}\frac{\partial }{\partial \alpha_{n}}\pm \alpha_{n}^{*}\frac{\partial }{\partial \alpha_{m}^{*}}, \end{array}$$

$n_{T}={\left(e^{z_{T}}-1\right)}^{-1}$ is the *mean number of thermal photons* and $\Gamma =(1-e^{-z_{T}})K$ is the *relaxation matrix* giving the rates of thermalization.

By using the method of characteristics to solve the initial value problem for the dynamical Equation (24) with the initial condition

(27) $$\begin{array}{cc} \; & \chi \left(\alpha ,0\right)\equiv \chi_{\mathrm{ini}}\left(\alpha \right)=e^{-{\left|\alpha \right|}^{2}/2}\chi_{N}^{\left(\mathrm{ini}\right)}\left(\alpha \right) \end{array}$$

we can derive the following expression for the normally ordered characteristic function [43,55]:

(28) $$\begin{array}{cc} \; & \chi_{N}\left(\alpha ,t\right)=e^{-(\alpha^{*},B\left(t\right)\alpha )}\chi_{N}^{\left(\mathrm{ini}\right)}\left(A\left(t\right)\alpha \right), \end{array}$$

(29) $$\begin{array}{cc} \; & A\left(t\right)=e^{(i\Omega -\Gamma )t},\;B\left(t\right)=n_{T}\left(I_{N}-A^{†}\left(t\right)A\left(t\right)\right), \end{array}$$

where $I_{N}$ is the identity N×N matrix. In Appendix B, this result is used to deduce the expression for the kernel of the Green’s function that determines time dependence of the Glauber–Sudarshan *P* function.

From Equation (28), it is not difficult to obtain the relations

(30) $$\begin{array}{cc} \; & \chi \left(\alpha ,t\right)=e^{-(\alpha^{*},C\left(t\right)\alpha )/2}\chi_{\mathrm{ini}}\left(A\left(t\right)\alpha \right), \end{array}$$

(31) $$\begin{array}{cc} \; & C\left(t\right)=\left(2n_{T}+1\right)\left(I_{N}-A^{†}\left(t\right)A\left(t\right)\right) \end{array}$$

describing the temporal evolution of the symmetrically ordered characteristic function, χ(α). This formula in combination with Equation (23) will be utilized in our subsequent calculations.

### 2.2. Evolution Speed of Gaussian States

In this section, we concentrate on the special case where the initial state is Gaussian and its characteristic function is of the following form

(32) $$\begin{array}{cc} \; & \chi_{\mathrm{ini}}\left(\alpha \right)=\exp\{-\frac{1}{2}\left(\alpha^{*},Q_{0}\alpha \right)\\ \; & +\frac{1}{4}\left[\left(\alpha ,P_{0}\alpha \right)+{\left(\alpha ,P_{0}\alpha \right)}^{*}\right]\}. \end{array}$$

By using Equation (30), the time dependence of the characteristic function

(33) $$\begin{array}{cc} \; & \chi \left(\alpha ,t\right)=\exp\{-\frac{1}{2}\left(\alpha^{*},Q\left(t\right)\alpha \right)\\ \; & +\frac{1}{4}\left[\left(\alpha ,P\left(t\right)\alpha \right)+{\left(\alpha ,P(t)\alpha \right)}^{*}\right]\} \end{array}$$

is expressed in terms of the two matrices

$$\begin{array}{cc} \; & Q\left(t\right)=\left(2n_{T}+1\right)I_{N} \end{array}$$

(34) $$\begin{array}{cc} \; & +A^{†}\left(t\right)\left[Q_{0}-\left(2n_{T}+1\right)I_{N}\right]A\left(t\right), \end{array}$$

(35) $$\begin{array}{cc} \; & P\left(t\right)=A^{T}\left(t\right)P_{0}A\left(t\right), \end{array}$$

where $A^{T}$ is the transpose of A. Similar to $Q_{0}$ and $P_{0}$, the matrices Q and P are Hermitian and symmetric, respectively.

The exponent on the right-hand side of Equation (33) can be conveniently cast into the real-valued form

(36) $$\begin{array}{cc} \; & \chi \left(s,t\right)=\exp\left\{-\frac{1}{4}\left(s,G\left(t\right)s\right)\right\}, \end{array}$$

(37) $$\begin{array}{cc} \; & G=\left(\begin{array}{cc} Q_{R}-P_{R} & -Q_{I}+P_{I}\\ Q_{I}+P_{I} & Q_{R}+P_{R} \end{array}\right), \end{array}$$

where α is replaced by $s=\left(x_{1},\ldots ,x_{N},p_{1},\ldots ,p_{N}\right)\equiv \left(x,p\right)$ with xi=2Reαi and pi=2Imαi. Note that in Equation (37), the subscripts “R” and “I” indicate the real and the imaginary parts of a matrix, respectively: AR=ReA and AI=ImA.

The 2×2 block-structure of the matrix (37) with rearranged elements reads

(38) $$\begin{array}{cc} \; & \tilde{G}=T^{T}GT,\;\left(\begin{array}{c} x_{1}\\ \vdots \\ x_{N}\\ p_{1}\\ \vdots \\ p_{N} \end{array}\right)=T\left(\begin{array}{c} x_{1}\\ p_{1}\\ \vdots \\ x_{N}\\ p_{N} \end{array}\right), \end{array}$$

where T is the permutation matrix, and it is closely related to the block structure of the covariance matrix, Σ, which is defined in terms of the quadrature operators (see, e.g., Refs. [45,57])

(39) $$\begin{array}{c} {\hat{x}}_{i}=\frac{\hat{a}+{\hat{a}}^{†}}{\sqrt{2}},\;{\hat{p}}_{i}=\frac{\hat{a}-{\hat{a}}^{†}}{\sqrt{2}i}. \end{array}$$

The relation linking the block matrices of Σ and $\tilde{G}$ reads

(40) $$\begin{array}{cc} \; & \Sigma_{ij}=\left(\begin{array}{cc} \langle \left\{{\hat{x}}_{i},{\hat{x}}_{j}\right\}\rangle & \langle \left\{{\hat{x}}_{i},{\hat{p}}_{j}\right\}\rangle \\ \langle \left\{{\hat{p}}_{i},{\hat{x}}_{j}\right\}\rangle & \langle \left\{{\hat{p}}_{i},{\hat{p}}_{j}\right\}\rangle \end{array}\right)-2\langle {\hat{r}}_{i}\rangle ⊗\langle {\hat{r}}_{j}\rangle \\ \; & =\sigma_{2}{\tilde{G}}_{ij}\sigma_{2}, \end{array}$$

where $\sigma_{2}$ is one of the Pauli matrices given by

(41) $$\begin{array}{cc} \; & \sigma_{1}=\left(\begin{array}{cc} 0 & 1\\ 1 & 0 \end{array}\right),\;\sigma_{2}=\left(\begin{array}{cc} 0 & -i\\ i & 0 \end{array}\right),\;\sigma_{3}=\left(\begin{array}{cc} 1 & 0\\ 0 & -1 \end{array}\right). \end{array}$$

Now, we pass on to computing of the generalized two-point fidelity given by Equation (3). To this end, we substitute the characteristic functions of the form (36) into the relation (23). After performing the resulting Gaussian integral, we derive the identity

(42) $$\begin{array}{cc} \; & F\left(t_{1},t_{2}\right)=\mathrm{Tr}\left(\hat{\rho }\left(t_{1}\right)\hat{\rho }\left(t_{2}\right)\right)=\frac{1}{{\left(2\pi \right)}^{N}}\int d\left(s\right)\chi \left(s,t_{1}\right)\chi \left(s,t_{2}\right)\\ \; & =2^{N}{\left[\det\left(G\left(t_{1}\right)+G\left(t_{2}\right)\right)\right]}^{-1/2}, \end{array}$$

where $d\left(s\right)=\prod_{i=1}^{N}dx_{i}dp_{i}$, giving the expressions for the purity (4)

(43) $$\begin{array}{cc} \; & P\left(t\right)=\mathrm{Tr}{\hat{\rho }}^{2}\left(t\right)\equiv F\left(t,t\right)={\left[\det G\left(t\right)\right]}^{-1/2} \end{array}$$

and for the fidelity of evolution (2)

(44) $$\begin{array}{c} F\left(t\right)=\mathrm{Tr}\left(\hat{\rho }\left(0\right)\hat{\rho }\left(t\right)\right)\equiv F\left(0,t\right)=2^{N}{\left[\det\left(G\left(0\right)+G\left(t\right)\right)\right]}^{-1/2}. \end{array}$$

We are also in a position to evaluate the squared speed of evolution by substituting derivatives of the determinant that enters Equation (42) with respect $t_{1}$ and $t_{2}$ into the relation

(45) $$\begin{array}{cc} \; & v^{2}\left(t\right)=\mathrm{Tr}{\left[\frac{\partial \rho (t)}{\partial t}\right]}^{2}\\ \; & =\frac{\partial^{2}}{\partial t_{1}\partial t_{2}}F\left(t_{1},t_{2}\right){|}_{t_{1}=t_{2}=t}=\frac{1}{16{\left(\det G\right)}^{5/2}}\\ \; & \times \left[3{\left(\frac{\partial \det G}{\partial t}\right)}^{2}-2\det G\mathrm{Tr}\left(\dot{G}{\dot{G}}^{\left(c\right)}\right)\right]. \end{array}$$

An alternative way to compute $v^{2}\left(t\right)$ is to evaluate the integral for the squared time derivative of the characteristic function

(46) $$\begin{array}{c} v^{2}\left(t\right)=\frac{1}{{\left(2\pi \right)}^{N}}\int d\left(s\right){\left[\frac{\partial \chi (s,t)}{\partial t}\right]}^{2}. \end{array}$$

It leads to the following formula for the squared speed of evolution:

(47) $$\begin{array}{cc} \; & v^{2}\left(t\right)=\frac{1}{16{\left(2\pi \right)}^{N}}\int d\left(s\right){\left(s,\dot{G}\left(t\right)s\right)}^{2}\exp\left\{-\frac{1}{2}\left(s,G\left(t\right)s\right)\right\}\\ \; & =\frac{1}{16\sqrt{\det G}}\left\{{\left[\mathrm{Tr}\left(\dot{G}G^{-1}\right)\right]}^{2}+2\mathrm{Tr}\left({\left[\dot{G}G^{-1}\right]}^{2}\right)\right\}. \end{array}$$

We conclude this section with the remark that the algebraic identities valid for a symmetric matrix G

(48) $$\begin{array}{cc} \; & \mathrm{Tr}\dot{G}G^{\left(c\right)}=\frac{\partial \det G}{\partial t}, \end{array}$$

(49) $$\begin{array}{cc} \; & G^{-1}\dot{G}G^{-1}={\left(\det G\right)}^{-1}\left\{\frac{\partial \det G}{\partial t}G^{-1}-{\dot{G}}^{\left(c\right)}\right\}, \end{array}$$

where $G^{\left(c\right)}=\left(\det G\right)G^{-1}$ is the matrix of cofactors, which can be used to show that differentiating the two-point fidelity (42) and computing traces that enter the last expression on the right-hand side of Equation (47) yield identical results for the evolution speed. With the help of Equation (43) giving the purity, the latter can be further simplified as follows:

(50) $$\begin{array}{cc} \; & v^{2}\left(t\right)=3{\left(\frac{\partial }{\partial t}\sqrt{P}\right)}^{2}-\frac{P}{8}\mathrm{Tr}\left(\dot{G}{\dot{G}}^{\left(c\right)}\right). \end{array}$$

## 3. Evolution of Two-Mode Systems

In this section, general results of the previous section will be applied to the special case with N=2. Thus, we focus our attention on the evolution of two-mode bosonic systems. As it was discussed above, such systems play an important part in the quantum optics as they represent polarization degrees of freedom of a quantized photonic mode.

### 3.1. Purity, Fidelity and Speed of Evolution

For the two-mode system, the frequency and relaxation matrices can be written as linear combinations of the Pauli matrices

(51) $$\begin{array}{cc} \; & i\Omega -\Gamma =\sum_{k=0}^{3}\left(i\omega_{k}-\gamma_{k}\right)\sigma_{k}=\left(i\omega_{0}-\gamma_{0}\right)\sigma_{0}\\ \; & +\left(i\omega -\gamma ,\sigma \right),\;\sigma_{0}\equiv I_{2} \end{array}$$

where $\sigma \equiv (\sigma_{1},\sigma_{2},\sigma_{3})$; $\omega \equiv (\omega_{1},\omega_{2},\omega_{3})$ and $\gamma \equiv (\gamma_{1},\gamma_{2},\gamma_{3})$ is the frequency and the relaxation rate vector, respectively. Thus, the dynamical interactions of the modes are described by the *frequency vector* ω, whereas the environment-mediated intermode couplings are determined by the *relaxation rate vector*, γ. These vectors that might be called the *intermode coupling vectors* govern the regime of dissipative dynamics and will be parameterized using the angular representation:

(52) $$\begin{array}{cc} \; & \gamma =\Gamma (\sin\theta_{\Gamma }\cos\phi_{\Gamma },\sin\theta_{\Gamma }\sin\phi_{\Gamma },\cos\theta_{\Gamma }),\\ \; & \omega =\Omega (\sin\theta_{\Omega }\cos\phi_{\Omega },\sin\theta_{\Omega }\sin\phi_{\Omega },\cos\theta_{\Omega }). \end{array}$$

It is rather straightforward to obtain the matrix exponential for A(t) (see Equation (29)) in the following explicit form:

(53) $$\begin{array}{cc} \; & A\left(t\right)=e^{(i\omega_{0}-\gamma_{0})t}\{\cosh\left(\left(i\omega -\gamma \right)t\right)\sigma_{0}\\ \; & +\frac{\sinh((i\omega -\gamma )t)}{\gamma -i\omega }\left(\gamma -i\omega ,\sigma \right)\}, \end{array}$$

where

(54) $$\begin{array}{cc} \; & n={\left(\gamma -i\omega \right)}^{-1}\left[\gamma -i\omega \right]=n_{R}+in_{I},\\ \; & \gamma -i\omega =\sqrt{\left(\gamma -i\omega ,\gamma -i\omega \right)}=\sqrt{\sum_{k=1}^{3}{\left(\gamma_{k}-i\omega_{k}\right)}^{2}}, \end{array}$$

where nR=Ren, nI=Imn, $|n_{R}{|}^{2}-{\left|n_{I}\right|}^{2}=1$, $n_{R}⊥n_{I}$.

We assume that the system is initially prepared in the two-mode squeezed state

(55) $$\begin{array}{c} |\psi_{0}\rangle ={|\eta \rangle }_{\mathrm{sq}}=\exp\left[\eta {\hat{K}}_{+}-\eta^{*}{\hat{K}}_{-}\right]|0\rangle ,\;\eta =re^{i\theta_{0}}, \end{array}$$

where *r* is the squeezing parameter, and ${\hat{K}}_{+}={\hat{a}}_{1}^{†}{\hat{a}}_{2}^{†}$ (${\hat{K}}_{-}={\hat{a}}_{1}{\hat{a}}_{2}$) is the raising (lowering) generator of the su(1,1) Lie algebra. For this state, the characteristic function is given by Equation (32) with the matrices

(56) $$\begin{array}{cc} \; & Q_{0}=\cosh\left(2r\right)\sigma_{0},\;P_{0}=\sinh\left(2r\right)e^{-i\theta_{0}}\sigma_{1}. \end{array}$$

For the evolved quantum state with the density matrix $\hat{\rho }\left(t\right)$, the explicit expression for the matrix Q(t) (see Equation (34)) that enters the characteristic function (33) is

(57) $$\begin{array}{cc} \; & Q\left(t\right)=\left(2n_{T}+1\right)\sigma_{0}+(\cosh\left(2r\right)\\ \; & -\left(2n_{T}+1\right))A^{†}\left(t\right)A\left(t\right), \end{array}$$

(58) $$\begin{array}{cc} \; & A^{†}\left(t\right)A\left(t\right)=\frac{e^{-2\gamma_{0}t}}{2}\left\{\cosh\left(2\gamma t\right)[(1+\right|n_{R}{|}^{2}+|n_{I}{|}^{2})\sigma_{0}\\ \; & -2\left(n_{R}\times n_{I},\sigma \right)]-2\sinh\left(2\gamma t\right)\left(n_{R},\sigma \right)+\cos\left(2\omega t\right)\\ \; & \times \left[\left(1-\right|n_{R}{|}^{2}-|n_{I}{|}^{2})\sigma_{0}+2\left(n_{R}\times n_{I},\sigma \right)\right]\\ \; & -2\sin\left(2\omega t\right)\left(n_{I},\sigma \right)\}, \end{array}$$

whereas the result for the matrix P(t) (see Equation (35)) is given by

(59) $$\begin{array}{cc} \; & P\left(t\right)=\sinh\left(2r\right)e^{-i\theta_{0}}A^{T}\left(t\right)\sigma_{1}A\left(t\right),\\ \; & A^{T}\left(t\right)\sigma_{1}A\left(t\right)=\frac{e^{2(i\omega_{0}-\gamma_{0})t}}{2}\sigma_{1}\{\left(\cosh2\left(i\omega -\gamma \right)t+1\right)\sigma_{0}\\ \; & +\left(\cosh2\left(i\omega -\gamma \right)t-1\right)\left[\left(1-2n_{3}^{2}\right)\sigma_{0}+2n_{3}\left(n_{⊥}\times e_{3},\sigma \right)\right] \end{array}$$

(60) $$\begin{array}{cc} \; & +2\left(n_{⊥},\sigma \right)\sinh2\left(i\omega -\gamma \right)t\}, \end{array}$$

where

(61) $$\begin{array}{c} n=n_{⊥}+n_{3}e_{3},\;n_{⊥}=\left(n_{1},n_{2},0\right). \end{array}$$

Our calculations can be divided into the three following steps: (a) formulas (57)–(60) are substituted into Equation (37) to obtain the matrix G; (b) by using this matrix, we compute the purity (43), the fidelity of evolution (44) and the speed of evolution (47); (c) the results are used to evaluate the QSL time for the HS distance (9) and the fidelity-based QSL time (10).

### 3.2. Mutual Information and Disentanglement Time

The covariance matrix Σ can also be readily computed from the matrix G. According to Equation (38), the matrix

(62) $$\begin{array}{cc} \; & \tilde{G}=\left(\begin{array}{cc} {\tilde{G}}_{11} & {\tilde{G}}_{12}\\ {\tilde{G}}_{12}^{T} & {\tilde{G}}_{22} \end{array}\right)=\mathrm{diag}\left(1,\sigma_{1},1\right)G\mathrm{diag}\left(1,\sigma_{1},1\right). \end{array}$$

gives the following block structure of the covariance matrix:

(63) $$\begin{array}{cc} \; & \Sigma =\left(\begin{array}{cc} \Sigma_{11} & \Sigma_{12}\\ \Sigma_{12}^{T} & \Sigma_{22} \end{array}\right),\;\Sigma_{ij}=\sigma_{2}{\tilde{G}}_{ij}\sigma_{2}. \end{array}$$

For Gaussian states, the symplectic eigenvalues of the covariance matrix (63) are given by [45,57]:

(64) $$\begin{array}{cc} \; & 2\mu_{\pm }^{2}=\Delta_{+}\pm \sqrt{\Delta_{+}^{2}-4\det\Sigma }, \end{array}$$

where $\Delta_{\pm }=\det\Sigma_{11}+\det\Sigma_{22}\pm 2\det\Sigma_{12}$, determine the entropy

(65) $$\begin{array}{cc} \; & S\left[\hat{\rho }\right]=-\mathrm{Tr}\hat{\rho }\log_{2}\hat{\rho }=\sum_{\nu =\pm }\{\frac{\mu_{\nu }+1}{2}\log_{2}\frac{\mu_{\nu }+1}{2}\\ \; & -\frac{\mu_{\nu }-1}{2}\log_{2}\frac{\mu_{\nu }-1}{2}\} \end{array}$$

and the mutual information

(66) $$\begin{array}{cc} \; & I\left(1;2\right)=S\left[{\hat{\rho }}_{1}\right]+S\left[{\hat{\rho }}_{2}\right]-S\left[\hat{\rho }\right]=\sum_{i=1}^{2}\{\frac{\mu_{i}+1}{2}\log_{2}\frac{\mu_{i}+1}{2}\\ \; & -\frac{\mu_{i}-1}{2}\log_{2}\frac{\mu_{i}-1}{2}\}-S\left[\hat{\rho }\right],\;\mu_{i}^{2}=\det\Sigma_{ii} \end{array}$$

where $\mu_{1}$ and $\mu_{2}$ are the one-mode symplectic eigenvalues for the reduced one-mode density matrices, ${\hat{\rho }}_{1}={\mathrm{Tr}}_{2}\hat{\rho }$ and ${\hat{\rho }}_{2}={\mathrm{Tr}}_{1}\hat{\rho }$, respectively.

Note that the mutual information can be defined as the relative entropy, $D(\hat{\rho }||{\hat{\rho }}_{1}⊗{\hat{\rho }}_{2})$, of the two-mode density matrix $\hat{\rho }$ to the product of the reduced one-mode density matrices ${\hat{\rho }}_{1}⊗{\hat{\rho }}_{2}$, which is an important entropic measure of the degree of correlation between the modes. An important point is that it contains the classical part of correlations and thus cannot be employed as an indicator of non-local quantum correlations such as entanglement.

For two-mode Gaussian states, different measures of entanglement have been proposed. These include the entanglement of formation, the Bures distance, and the Gaussian measures of entanglement [59,60,61]. For our purposes, similar to [43], it is convenient to deploy a quantifier of bipartite entanglement in Gaussian states which is expressed in terms of the lowest symplectic eigenvalue, $\lambda_{PT}$, of the partial transpose of the two-mode density matrix, ${\hat{\rho }}^{PT}$, which is given by

(67) $$\begin{array}{cc} \; & 2\lambda_{PT}^{2}=\Delta_{-}-\sqrt{\Delta_{-}^{2}-4\det\Sigma }. \end{array}$$

One of such quantifiers is the logarithmic negativity [57]

(68) $$\begin{array}{c} E_{N}\left(\hat{\rho }\right)=\log_{2}\left|\right|{\hat{\rho }}^{PT}{\left|\right|}_{1}=\max\left\{0,-\log_{2}\lambda_{PT}\right\}. \end{array}$$

It implies that the entangled states meet the condition: $\lambda_{PT}<1$ and, in the regime of finite-time disentanglement, the solution of equation

(69) $$\begin{array}{c} \lambda_{PT}\left(t_{d}\right)=1 \end{array}$$

gives the disentanglement time $t_{d}$.

## 4. Results

As we have seen, for the two-mode systems, dynamical and environment-mediated intermode interactions appear to be encoded into the frequency and the relaxation vectors, ω and γ. Thus, we can expect that temporal evolution will crucially depend on the orientation of these vectors. According to Refs. [28,43], the angle between ω and γ is one of the important factors determining dynamical regimes of depolarization and disentanglement. In order to understand the orientation-dependent effects, we begin with the simplest limiting case where γ‖ω and iω−γ=iΩ−Γ.

### 4.1. Dynamical Regimes at γ ‖ ω and γ ⊥ ω

To be more specific, we assume that the vectors are oriented along the $e_{3}$ axis, $i\omega -\gamma =\left(i\Omega -\Gamma \right)e_{3}$, so that the matrices iΩ−Γ and A(t) are both diagonal. After evaluating the matrices Q and P (see Equations (57) and (59))

(70) $$\begin{array}{cc} \; & Q=\mathrm{diag}\left(q_{1},q_{2}\right),\;q_{1,2}=q_{0}e^{-2(\gamma_{0}\pm \Gamma )t}+\left(1+2n_{T}\right),\\ \; & q_{0}=\cosh\left(2r\right)-1-2n_{T},\\ \; & P=pe^{-i\theta }\sigma_{1},\;p=\sinh\left(2r\right)e^{-2\gamma_{0}t},\;\theta =\theta_{0}-2\omega_{0}t \end{array}$$

we obtain the matrices G (see Equation (37)) and $\tilde{G}$ (see Equation (62)). The latter is described by the 2×2 block matrices

(71) $$\begin{array}{c} {\tilde{G}}_{ii}=q_{i}\sigma_{0},\;{\tilde{G}}_{12}=p\left(\begin{array}{cc} -\cos\theta & -\sin\theta \\ -\sin\theta & \cos\theta \end{array}\right) \end{array}$$

that determine the block structure of the covariance matrix (63).

The two-point fidelity (42) can now be derived as an explicit function of $t_{1}$ and $t_{2}$ of the following form:

(72) $$\begin{array}{cc} \; & F\left(t_{1},t_{2}\right)=\mathrm{Tr}\left(\hat{\rho }\left(t_{1}\right)\hat{\rho }\left(t_{2}\right)\right)=\frac{4}{{\tilde{q}}_{1}{\tilde{q}}_{2}-{\tilde{p}}^{2}},\\ \; & {\tilde{q}}_{i}=q_{i}\left(t_{1}\right)+q_{i}\left(t_{2}\right),\;{\tilde{p}}^{2}=p^{2}\left(t_{1}\right)+p^{2}\left(t_{2}\right)\\ \; & +2p\left(t_{1}\right)p\left(t_{2}\right)\cos\left(\theta \left(t_{1}\right)-\theta \left(t_{2}\right)\right). \end{array}$$

Then, the relations

(73) $$\begin{array}{cc} \; & P\left(t\right)=F\left(t,t\right)=\frac{1}{q_{1}\left(t\right)q_{2}\left(t\right)-p^{2}\left(t\right)},\\ \; & F(t)=F(0,t) \end{array}$$

give the purity (43) and the fidelity of evolution (44).

It is now rather straightforward to evaluate the derivatives (45) of the fidelity (72) with respect to $t_{1}$ and $t_{2}$ and deduce the following closed-form formula for the speed of evolution:

(74) $$\begin{array}{cc} \; & v\left(t\right)=\frac{1}{\sqrt{2}{\left(q_{1}q_{2}-p^{2}\right)}^{3/2}}\{{\left[\frac{\partial (q_{1}q_{2}-p^{2})}{\partial t}\right]}^{2}\\ \; & -\left[{\dot{q}}_{1}{\dot{q}}_{2}-{\left(\dot{p}\right)}^{2}-p^{2}{\left(\dot{\theta }\right)}^{2}\right]\left(q_{1}q_{2}-p^{2}\right){\}}^{1/2}. \end{array}$$

Since $\det\Sigma_{ij}=\det{\tilde{G}}_{ij}$ and $\Delta_{\pm }=q_{1}^{2}+q_{2}^{2}\mp 2p^{2}$, it is not difficult to obtain the symplectic eigenvalues (64)

(75) $$\begin{array}{cc} \; & 2\mu_{\pm }^{2}=q_{1}^{2}+q_{2}^{2}-2p^{2}\\ \; & \pm |q_{1}-q_{2}|\sqrt{{\left(q_{1}+q_{2}\right)}^{2}-4p^{2}}, \end{array}$$

and the lowest eigenvalue for the partial transpose of the density matrix (67)

(76) $$\begin{array}{cc} \; & 2\lambda_{PT}^{2}=q_{1}^{2}+q_{2}^{2}+2p^{2}\\ \; & -\left(q_{1}+q_{2}\right)\sqrt{{\left(q_{1}-q_{2}\right)}^{2}+4p^{2}}. \end{array}$$

In the long-time limit, all these eigenvalues approach the limiting value

(77) $$\begin{array}{cc} \; & \mu_{\pm }\left(\infty \right)=\lambda_{PT}\left(\infty \right)=1+2n_{T}={\left[\tanh\left(z_{T}/2\right)\right]}^{-1} \end{array}$$

that, similar to the steady-state density matrix: $\hat{\rho }\left(\infty \right)={\hat{\rho }}_{\mathrm{eq}}\propto \exp\left[-z_{T}\left({\hat{a}}_{1}^{†}{\hat{a}}_{1}+{\hat{a}}_{2}^{†}{\hat{a}}_{2}\right)\right]$, is determined by the inverse temperature parameter $z_{T}$. Hence, at non-vanishing temperatures with $n_{T}>0$, the regime of finite-time disentanglement takes place, and the disentanglement time can be found by solving Equation (69).

At $\Gamma =\gamma_{3}=0$, we have $q_{1}=q_{2}=q$ and the simplified expression for $\lambda_{PT}$: $\lambda_{PT}=\left|p-q\right|$, gives the solution in the closed-form as follows

(78) $$\begin{array}{c} t_{d}\left(0\right)\equiv t_{d}{|}_{\Gamma =0}=\gamma_{0}^{-1}\ln\left(1+\frac{1-e^{-2r}}{2n_{T}}\right). \end{array}$$

This result was originally reported in [33].

**Figure 1:**
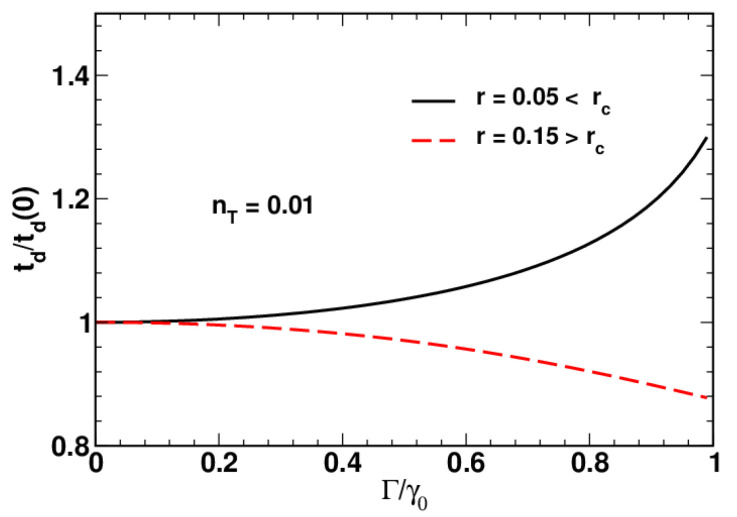
Disentanglement time ratio, $t_{d}/t_{d}\left(0\right)$, as a function of the relaxation rate ratio, $\Gamma /\gamma_{0}$, computed at different values of the squeezing parameter, *r*, for $n_{T}=0.01$.

Figure 1 illustrates the behavior of the disentanglement time, $t_{d}$, as the relaxation rate, Γ, increases approaching its limiting value $\gamma_{0}$. It can be seen that when the squeezing parameter *r* is sufficiently high, the disentanglement time decreases with Γ, whereas an increase in Γ will reduce the time $t_{d}$ provided the parameter *r* is below its critical value, $r_{c}$.

**Figure 2:**
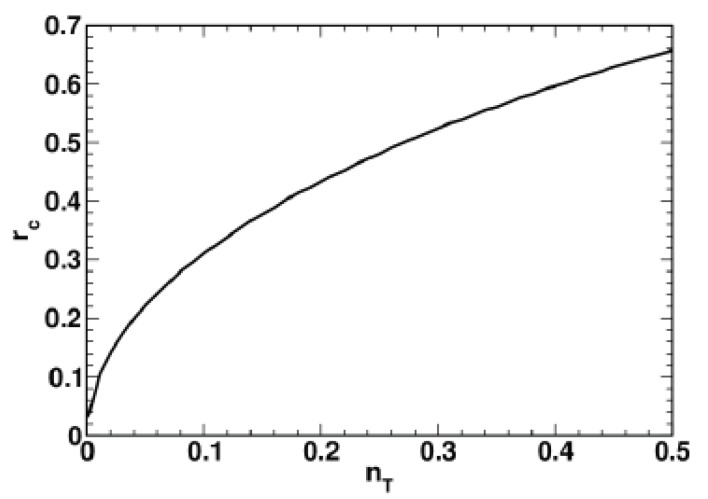
Critical value of the squeezing parameter, $r_{c}$, as a function of the mean number of thermal photons, $n_{T}={\left(e^{z_{T}}-1\right)}^{-1}$.

The critical value of the squeezing parameter, $r_{c}$, is plotted against the mean number of thermal photons, $n_{T}$, in Figure 2. From the $r_{c}$ vs. $n_{T}$ curve depicted in Figure 2, it can be inferred that the critical squeezing parameter grows with temperature.

**Figure 3:**
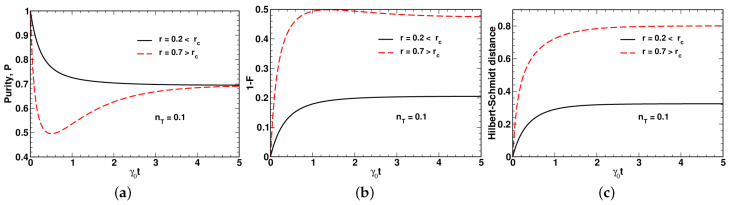
Temporal evolution of (**a**) purity, P(t), (**b**) change of fidelity, 1−F(t), and (**c**) HS distance, $∥\hat{\rho }\left(t\right)-\hat{\rho }{\left(0\right)∥}_{2}$, computed at different values of the squeezing parameter, *r*, for $n_{T}=0.1$, $\Gamma /\gamma_{0}=0.5$ and $\omega_{0}=0$.

As is shown in Figure 3a,b, the time dependencies of the purity (43) and the fidelity of evolution (44) (see also Equation (73)) reveal non-monotonic behavior when the squeezing parameter exceeds the threshold $r_{c}$. By contrast, Figure 3c shows that the HS distance is a monotonically increasing function of time that in the long-time limit with t→∞, approaches its asymptotic value:

(79) $$\begin{array}{c} ∥{\hat{\rho }}_{\mathrm{eq}}-\hat{\rho }{\left(0\right)∥}_{2}=\sqrt{1-2F(\infty )+P(\infty )}, \end{array}$$

where F(∞) and P(∞) are the long-time asymptotics for the fidelity and the purity given by

(80) $$\begin{array}{cc} \; & F\left(\infty \right)={\left(\cosh^{2}\left(r\right)\left(1+2n_{T}\right)+n_{T}^{2}\right)}^{-1},\\ \; & P\left(\infty \right)={\left(1+2n_{T}\right)}^{-2}. \end{array}$$

The long-time behavior of the HS distance and the fidelity-based QSL times, $t_{HS}$ and $t_{F}$, (see Equations (9) and (10)), is determined by the coefficients

(81) $$\begin{array}{cc} \; & t_{HS}/t\approx \kappa_{HS}=\frac{∥{\hat{\rho }}_{\mathrm{eq}}-\hat{\rho }{\left(0\right)∥}_{2}}{s_{\infty }},\\ \; & t_{F}/t\approx \kappa_{F}=\frac{1-F(\infty )}{s_{\infty }},\;s_{\infty }=\int_{0}^{\infty }v\left(t\right)dt \end{array}$$

that might be called the QSL long-time asymptotic ratios. Typically, the evolution speed decays rapidly with time, and thus, the nonlinear corrections for the time dependence of QSL times are small. So, we concentrate on the QSL time ratios, $\kappa_{HS}$ and $\kappa_{F}$.

**Figure 4:**
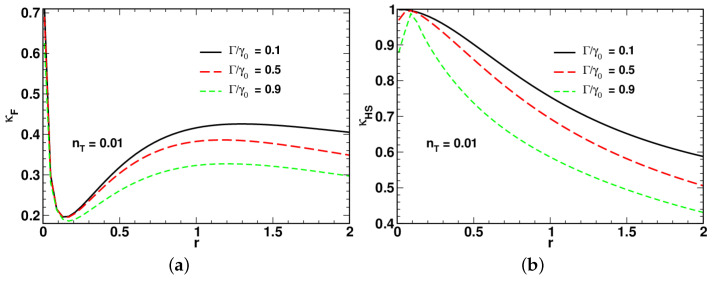
Dependence of long-time asymptotic ratios for (**a**) fidelity-based and (**b**) HS distance-based QSL times, $t_{F}$ (see Equation (10)) and $t_{HS}$ (see Equation (9)), on the squeezing parameter computed at different values of the relaxation rate ratio $\Gamma /\gamma_{0}$ for $n_{T}=0.01$ and $\omega_{0}=0$.

**Figure 5:**
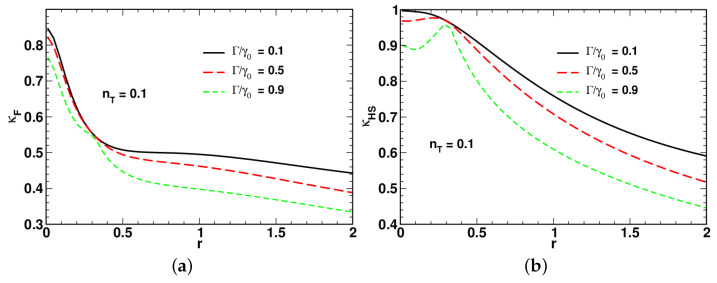
Dependence of long-time asymptotic ratios for (**a**) fidelity-based and (**b**) HS distance-based QSL times, $t_{F}$ (see Equation (10)) and $t_{HS}$ (see Equation (9)), on the squeezing parameter computed at different values of the relaxation rate ratio $\Gamma /\gamma_{0}$ for $n_{T}=0.1$ and $\omega_{0}=0$.

**Figure 6:**
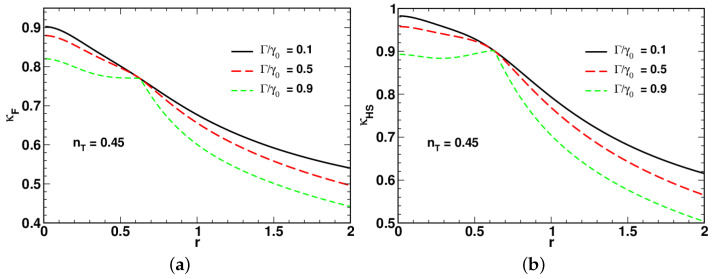
Dependence of long-time asymptotic ratios for (**a**) fidelity-based and (**b**) HS distance-based QSL times, $t_{F}$ (see Equation (10)) and $t_{HS}$ (see Equation (9)), on the squeezing parameter computed at different values of the relaxation rate ratio $\Gamma /\gamma_{0}$ for $n_{T}=0.45$ and $\omega_{0}=0$.

Figure 4, Figure 5 and Figure 6 present the results for dependencies of the long-time asymptotic ratios on the squeezing parameter computed at different values of the relaxation rate and the mean number of thermal photons.

Referring to Figure 4, the low-temperature curves for the ratio of the fidelity-based QSL time plotted in Figure 4a demonstrate non-monotonic behavior with a pronounced minimum. By contrast, the curves for the long-time ratio $\kappa_{HS}$ shown in Figure 4b, after reaching a local maximum in the low squeezing region, decline as the squeezing parameter *r* increases.

In Figure 4, Figure 5 and Figure 6, an increase in the relaxation rate Γ leads to reduction of both the ratios $\kappa_{HS}$ and $\kappa_{F}$. An additional effect of the relaxation rate is to enhance the non-monotonic behavior of $\kappa_{HS}$ in the low squeezing region (see Figure 5b).

A comparison between the results depicted in Figure 4a and Figure 5a shows that the minimum is suppressed at elevated temperature. Interestingly, as is shown in Figure 6, in the vicinity of the high-temperature limit with $n_{T}=0.5$, the curves for $\kappa_{HS}$ and $\kappa_{F}$ become remarkably similar. When the relaxation rate Γ is close to $\gamma_{0}$, the shape of the squeezing parameter dependence of the long-time ratios reveals two dynamical regimes that take place in the well-separated low-squeezing and high-squeezing regions.

### 4.2. Dynamical Regimes at γ ⊥ ω

We conclude our discussion of the dynamical regimes with remarks about another limiting case where the relaxation vector γ is reoriented along the normal to the frequency vector ω as follows

(82) $$\begin{array}{c} \gamma -i\omega =\left(\Gamma ,0,-i\Omega \right),\;\gamma -i\omega =\sqrt{\Gamma^{2}-\Omega^{2}}. \end{array}$$

In this case, the matrix P (see Equation (59)) can be cast into the form:

(83) $$\begin{array}{c} P\left(t\right)=\sinh\left(2r\right)e^{-i\theta }\sigma_{1}A^{†}\left(t\right)A\left(t\right), \end{array}$$

where, similar to the matrix Q given by Equation (57), the time-dependent part is solely determined by the matrix product $A^{†}\left(t\right)A\left(t\right)$.

Therefore, it might be expected that the time dependence of $A^{†}\left(t\right)A\left(t\right)$ dictates the dynamical regimes. According to Equation (82), similar to the simple PT-symmetric two-level system studied in [16], the value of γ−iω is either real or imaginary depending on the sign of the difference between Γ and |Ω|.

When Γ>Ω and ω=0 ($\gamma =\sqrt{\Gamma^{2}-\Omega^{2}}$), the expression for $A^{†}\left(t\right)A\left(t\right)$ is

(84) $$\begin{array}{cc} \; & A^{†}\left(t\right)A\left(t\right)=\frac{e^{-2\gamma_{0}t}}{\gamma^{2}}\{\left[\Gamma^{2}\cosh\left(2\gamma t\right)-\Omega^{2}\right]\sigma_{0}\\ \; & -\Gamma \Omega \left[\cosh(2\gamma t)-1\right]\sigma_{2}-\Gamma \gamma \sinh\left(2\gamma t\right)\sigma_{1}\}. \end{array}$$

By contrast, in the opposite case with Γ<Ω and γ=0 ($\omega =\sqrt{\Omega^{2}-\Gamma^{2}}$), the result

(85) $$\begin{array}{cc} \; & A^{†}\left(t\right)A\left(t\right)=\frac{e^{-2\gamma_{0}t}}{\omega^{2}}\{\left[\Omega^{2}-\Gamma^{2}\cos\left(2\omega t\right)\right]\sigma_{0}\\ \; & +\Gamma \Omega \left[\cos(2\omega t)-1\right]\sigma_{2}-\Gamma \omega \sin\left(2\omega t\right)\sigma_{1}\} \end{array}$$

describes an alternative regime associated with the presence of oscillations.

Finally, the critical regime that occurs when Γ=|Ω| and ω=γ=0 is represented by the matrix

(86) $$\begin{array}{cc} \; & A^{†}\left(t\right)A\left(t\right)=e^{-2\gamma_{0}t}\{[1+2{\left(\Gamma t\right)}^{2}]\sigma_{0}\\ \; & -2{\left(\Gamma t\right)}^{2}\sigma_{2}-2\Gamma t\sigma_{1}\}. \end{array}$$

This expression implies the dissipative dynamics will start to slow down when the oscillatory regime is changed to the critical one.

### 4.3. Effects of Intermode Couplings

As it was mentioned above, the evolution speed typically evolves monotonically approaching zero and the speed evaluated at initial instant of time, t=0, gives its maximum value. This value is one of the factors that influence the QSL times and dynamical regimes.

**Figure 7:**
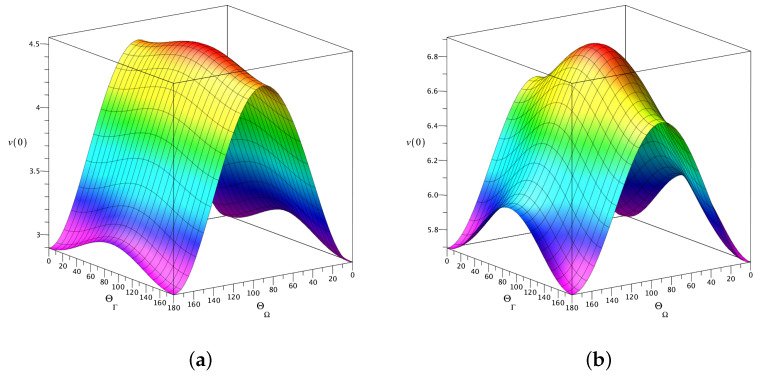
Angular dependence of initial speed of evolution, v(0), computed at (**a**) $n_{T}=0.1$ and (**b**) $n_{T}=0.45$ with $\Gamma /\gamma_{0}=0.5$. Other parameters are: r=0.5, $\Omega /\gamma_{0}=2.0$, $\omega_{0}=0$ and $\theta_{0}=\phi_{\Gamma }=\phi_{\Omega }=0$.

**Figure 8:**
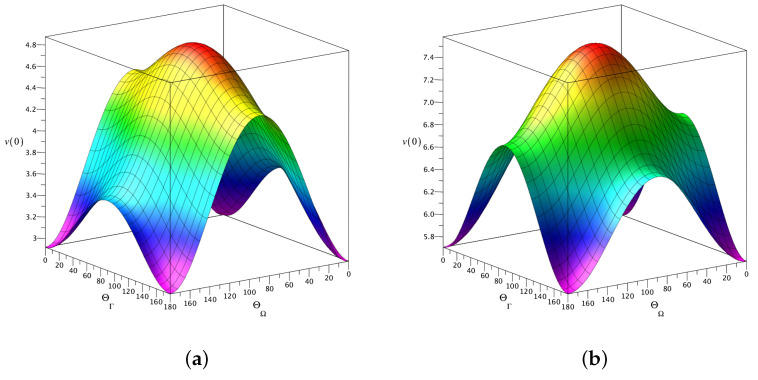
Angular dependence of initial speed of evolution, v(0), computed at (**a**) $n_{T}=0.1$ and (**b**) $n_{T}=0.45$ with $\Gamma /\gamma_{0}=0.9$. The parameters are listed in the caption of Figure 7.

Figure 7 and Figure 8 show how the maximum value of the evolution speed depends on the orientation of the frequency and relaxation vectors in the $e_{1}$–$e_{3}$ plane. The surfaces plotted in Figure 7 and Figure 8 represent angular distributions for v(0) computed as a function of the polar angles $\theta_{\Gamma }$ and $\theta_{\Omega }$ at the two values of the relaxation rate ratio: $\Gamma /\gamma_{0}=0.5$ and $\Gamma /\gamma_{0}=0.9$, respectively. For both values of the relaxation rate, we have evaluated the low-temperature ($n_{T}=0.1$) and high-temperature ($n_{T}=0.45$) angular distributions.

At $\Gamma /\gamma_{0}=0.5$, referring to Figure 7a, it can be seen that the depth of modulation of the speed v(0) induced variations in the angle $\theta_{\Gamma }$ is noticeably smaller than that induced by the angle $\theta_{\Omega }$. As is shown in Figure 7b, an increase in temperature will raise the speed and make the $\theta_{\Gamma }$-induced modulation of v(0) more pronounced.

When the relaxation rate is increased up to $\Gamma /\gamma_{0}=0.9$ (see Figure 8), we arrive at similar conclusions. In this case, both the temperature-induced speed up of the evolution and the $\theta_{\Gamma }$-induced modulation of v(0) are noticeably stronger as compared to the results presented in Figure 7.

We have also computed time dependencies of the entropy and the mutual information for differently oriented vectors γ and ω. The list of parameters used in these calculations is as follows: $n_{T}=0.1$, r=2.0, $\Gamma /\gamma_{0}=0.9$ and $\theta =\phi_{\Gamma }=\phi_{\Omega }=0$. At these parameters, we have the initial values of the symplectic eigenvalues $\mu_{\pm }\left(0\right)=1$, $\mu_{1,2}\left(0\right)=\cosh\left(2r\right)$ and $\lambda_{PT}\left(0\right)=e^{-2r}$ giving the corresponding values for the entropy, $S\left(\hat{\rho }\right){|}_{t=0}=0$, and the mutual information, ${I\left(1;2\right)|}_{t=0}\approx 10.43$.

**Figure 9:**
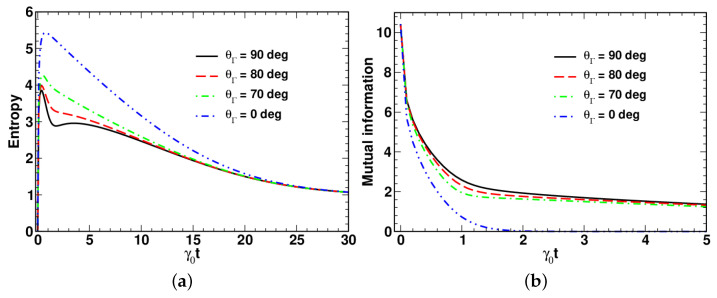
Temporal evolution of (**a**) entropy and (**b**) mutual information computed at different values of the angle $\theta_{\Gamma }$ with $\gamma =\Gamma (\sin\theta_{\Gamma },0,\cos\theta_{\Gamma })$ at $\Gamma /\gamma_{0}=0.9$, $\Omega /\gamma_{0}=0$, $n_{T}=0.1$, r=2 and $\theta_{0}=0$.

The results for the entropy and the mutual information are depicted in Figure 9a,b, respectively. As is shown in Figure 9b, the mutual information monotonically decays, whereas the time dependence of the entropy (see Figure 9a) is non-monotonic. It starts from zero and reaches its maximum value, which is well above its equilibrium value $S_{\mathrm{eq}}$.

**Figure 10:**
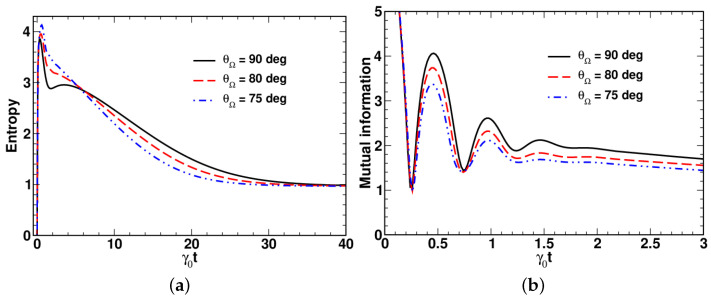
Temporal evolution of (**a**) entropy and (**b**) mutual information computed at different values of the angle $\theta_{\Omega }$ with $\omega =\Omega (\sin\theta_{\Omega },0,\cos\theta_{\Omega })$ for $\Gamma /\gamma_{0}=0.9$, $\theta_{\Gamma }=90$${}^{\circ }$, $\Omega /\gamma_{0}=\pi$, $n_{T}=0.1$, r=2 and $\theta_{0}=0$.

Referring to Figure 10, by contrast to the entropy curves presented in Figure 10a, the time dependence of the mutual information shown in Figure 10b exhibits pronounced oscillations induced by the intermode coupling.

## 5. Conclusions

In this paper, we have adopted a QSL-based approach to dynamics of a multi-mode bosonic system governed by the thermal bath Lindblad master equation. Within this approach, we have focused our attention on the speed of evolution and related time-dependent QSL times. It is found that the evolution speed gives the upper bound for the rate of change of both the fidelity and the Hilbert–Schmidt distance (see Equations (6) and (8)). So, the HS distance and the fidelity-based QSL times, $t_{HS}$ and $t_{F}$, (see Equations (9) and (10)) are both inversely proportional to the time average of the evolution speed.

By using exact solution for the characteristic function obtained in [43] (see Equations (28)–(31)), we have derived general formulas for the evolution speed (see Equations (45), (47) and (50)) and the QSL times of evolving Gaussian states. Note that the latter also involves explicit expressions for the purity (43) and the fidelity (44) giving the HS distance: $∥\hat{\rho }\left(t\right)-\hat{\rho }{\left(0\right)∥}_{2}=\sqrt{1-2F(t)+P(t)}$.

General analytical results are applied to the special case of the two-mode bosonic system. In this case, the dynamics is determined by the intermode couplings that enter the dynamical and relaxation parts of the Lindblad superoperator L (see Equation (13)) and can be conveniently described in terms of the frequency and the relaxation rate vectors (see Equation (51)), ω and γ, that might be referred to as the intermode coupling vectors.

For the system initially prepared in the two-mode squeezed state, we have analyzed dynamical regimes assuming that the intermode coupling vectors are parallel, $\omega \|\gamma \|e_{3}$. In general, these regimes depend on a number of factors such as the squeezing parameter, the intermode interactions and temperature.

It turned out that different regimes can be related to the disentanglement time, $t_{d}$, (see Equations (69) and (76)) which is found to either increase or decrease with the relaxation rate, Γ, equal to the magnitude of the relaxation vector depending on the value of the squeezing parameter (see Figure 1). The critical value of the squeezing parameter, $r_{c}$, separating the low-squeezing region, where $t_{d}$ grows with Γ, from the high-squeezing one is found to be an increasing function of temperature (see Figure 2). (In this paper, we use the temperature-dependent mean value of thermal photons $n_{T}$ instead of temperature.) Figure 3 shows that in the region above the threshold $r_{c}$, the time dependence of the purity and the fidelity becomes non-monotonic, whereas this is not the case for the HS distance.

The starting point of our study of the regimes associated with the QSL times was the long-time asymptotic behavior of the time-dependent QSL times characterized by the proportionality coefficients, $\kappa_{HS}$ and $\kappa_{F}$, that enter the linear relations linking the QSL times, $t_{HS}$ and $t_{F}$, and the actual time *t* (see Equation (81)). These coefficients called the large-time asymptotic ratios are plotted against the squeezing parameter at varying temperature and the relaxation rate Γ in Figure 4, Figure 5 and Figure 6. Interestingly, the curves for $\kappa_{HS}$ with Γ close to the overall relaxation rate $\gamma_{0}$ reveal a well-pronounced local maximum in the vicinity of the above squeezing threshold $r_{c}$. Note also that at high temperature, the curves for $\kappa_{HS}$ and $\kappa_{F}$ are similar in shape (see Figure 6). Referring to Figure 4, this is clearly no longer the case when the temperature is low.

For mutually orthogonal intermode coupling vectors, according to Equations (84)–(86), the dynamical regimes are dictated by the sign of the difference between the vector magnitudes: Γ−Ω. In a more general case, where the vectors are arbitrarily oriented in the $e_{1}$–$e_{3}$ plane, we have evaluated the maximum speed of evolution in relation to the polar angles $\theta_{\Gamma }$ and $\theta_{\Omega }$ (angular parametrization of the vectors is given by Equation (52)). The surfaces representing angular distributions of this speed are plotted in Figure 7Figure 8. These distributions demonstrate that the orientation of the intermode coupling vectors has a profound effect on the evolution speed.

Similar to the evolution speed, from Figure 9 and Figure 10, it can be inferred that the temporal evolution of the entropy and the mutual information is sensitive to variations in the polar angles. Note that the dynamics of the mutual information, I(1;2), qualitatively resembles the dynamical behavior of the logarithmic negativity, $E_{N}$, previously reported in [43].

The difference is that the decay rate of I(1;2) is typically noticeably slower as compared to the decay rate of $E_{N}$. In addition, $E_{N}$ vanishes at $t>t_{d}$, whereas the mutual information falls off approaching zero in the long-time limit, t→∞. These differences can be attributed to both the classical part of intermode correlations quantified by I(1;2) and the nonclassical correlations that differ from entanglement (such correlations can be characterized using the quantum discord that describes the nonlocal fraction of I(1;2)).

We conclude with the remark that our analytical approach provides tools for a more comprehensive analysis of the evolution speed and QSL-related problems for continuous variable systems in a realistic setup involving open environments and interacting modes. We hope that our work will stimulate further progress in the field.

## Data Availability

Not applicable.

## Appendix A. Liouvillian and Its Conjugate

In this appendix, our starting point is the relation

(A1) $$\begin{array}{cc} \; & \mathrm{Tr}\left\{\hat{O}L\left(\hat{\rho }\right)\right\}=\mathrm{Tr}\left\{L^{†}\left(\hat{O}\right)\hat{\rho }\right\} \end{array}$$

that introduces the superoperator $L^{†}$ as the adjoint (conjugate) of the Liouvillian superoperator, L, given by Equation (13). From this relation, the conjugates of the commutator (14) and the dissipator (15) are given by

(A2) $$\begin{array}{cc} \; & C_{\hat{A}\hat{B}}^{†}=-C_{\hat{A}\hat{B}},\;D_{\hat{A}\hat{B}}^{†}=2\hat{B}\hat{\rho }\hat{A}-\left\{\hat{B}\hat{A},\hat{\rho }\right\}. \end{array}$$

Algebraic identities for superoperators that enter both the commutators and the dissipators on the right-hand side of Equation (13)

(A3) $$\begin{array}{cc} \; & {\hat{a}}_{n}^{†}{\hat{a}}_{m}\hat{O}={\hat{a}}_{n}^{†}\left[{\hat{a}}_{m},\hat{O}\right]+{\hat{a}}_{n}^{†}\hat{O}{\hat{a}}_{m}, \end{array}$$

(A4) $$\begin{array}{cc} \; & \hat{O}{\hat{a}}_{n}^{†}{\hat{a}}_{m}=-\left[{\hat{a}}_{n}^{†},\hat{O}\right]{\hat{a}}_{m}+{\hat{a}}_{n}^{†}\hat{O}{\hat{a}}_{m},\\ \; & {\hat{a}}_{m}\hat{O}{\hat{a}}_{n}^{†}={\hat{a}}_{n}^{†}\left[{\hat{a}}_{m},\hat{O}\right]-\left[{\hat{a}}_{n}^{†},\hat{O}\right]{\hat{a}}_{m}-\left[{\hat{a}}_{n}^{†},\left[{\hat{a}}_{m},\hat{O}\right]\right] \end{array}$$

(A5) $$\begin{array}{cc} \; & +\delta_{mn}\hat{O}+{\hat{a}}_{n}^{†}\hat{O}{\hat{a}}_{m}, \end{array}$$

where $\hat{O}$ denotes an arbitrary operator, give the normally ordered form of the image. For the normally ordered displacement operator (17), it is not difficult to check the validity of the following relations

(A6) $$\begin{array}{cc} \; & \left[{\hat{a}}_{m},{\hat{D}}_{N}\right]=\alpha_{m}{\hat{D}}_{N},\;\left[{\hat{a}}_{n}^{†},{\hat{D}}_{N}\right]=\alpha_{n}^{*}{\hat{D}}_{N}, \end{array}$$

(A7) $$\begin{array}{cc} \; & {\hat{a}}_{n}^{†}{\hat{D}}_{N}=\frac{\partial }{\partial \alpha_{n}}{\hat{D}}_{N},\;{\hat{D}}_{N}{\hat{a}}_{m}=-\frac{\partial }{\partial \alpha_{m}^{*}}{\hat{D}}_{N}. \end{array}$$

From Equations (A3)–(A5), we can deduce formulas

$$\begin{array}{cc} \; & D_{{\hat{a}}_{m}{\hat{a}}_{n}^{†}}\hat{O}={\hat{a}}_{n}^{†}\left[{\hat{a}}_{m},\hat{O}\right]-\left[{\hat{a}}_{n}^{†},\hat{O}\right]{\hat{a}}_{m} \end{array}$$

(A8) $$\begin{array}{cc} \; & -2\left[{\hat{a}}_{n}^{†},\left[{\hat{a}}_{m},\hat{O}\right]\right]+2\delta_{mn}\hat{O}, \end{array}$$

(A9) $$\begin{array}{cc} \; & D_{{\hat{a}}_{m}{\hat{a}}_{n}^{†}}^{†}\hat{O}=-\left({\hat{a}}_{n}^{†}\left[{\hat{a}}_{m},\hat{O}\right]-\left[{\hat{a}}_{n}^{†},\hat{O}\right]{\hat{a}}_{m}\right), \end{array}$$

(A10) $$\begin{array}{cc} \; & D_{{\hat{a}}_{n}^{†}{\hat{a}}_{m}}\hat{O}=-\left({\hat{a}}_{n}^{†}\left[{\hat{a}}_{m},\hat{O}\right]-\left[{\hat{a}}_{n}^{†},\hat{O}\right]{\hat{a}}_{m}+2\delta_{mn}\hat{O}\right),\\ \; & D_{{\hat{a}}_{n}^{†}{\hat{a}}_{m}}^{†}\hat{O}={\hat{a}}_{n}^{†}\left[{\hat{a}}_{m},\hat{O}\right]-\left[{\hat{a}}_{n}^{†},\hat{O}\right]{\hat{a}}_{m} \end{array}$$

(A11) $$\begin{array}{cc} \; & -2[{\hat{a}}_{n}^{†},\left[{\hat{a}}_{m},\hat{O}\right]], \end{array}$$

giving the normally ordered images of $\hat{O}$ under the action of the dissipators and their conjugates.

By substituting $\hat{O}={\hat{D}}_{N}$ into Equations (A8)–(A11), and by using the relations

(A12) $$\begin{array}{cc} \; & {\hat{a}}_{n}^{†}\left[{\hat{a}}_{m},{\hat{D}}_{N}\right]=\alpha_{m}\frac{\partial }{\partial \alpha_{n}}{\hat{D}}_{N},\\ \; & -\left[{\hat{a}}_{n}^{†},{\hat{D}}_{N}\right]{\hat{a}}_{m}=\alpha_{m}^{*}\frac{\partial }{\partial \alpha_{m}^{*}}{\hat{D}}_{N},\\ \; & \left[{\hat{a}}_{n}^{†},\left[{\hat{a}}_{m},{\hat{D}}_{N}\right]\right]=\alpha_{m}\alpha_{n}^{*}{\hat{D}}_{N}, \end{array}$$

that follows from Equations (A6) and (A7), we obtain the differential operator (25) that governs the dynamical equation for the normally ordered characteristic function $\chi_{N}$.

Our concluding remark is that in the relation

(A13) $$\begin{array}{cc} \; & \left(L+L^{†}\right)\hat{O}=2\mathrm{Tr}\Gamma \;\hat{O}\\ \; & -\frac{2}{\tanh(z_{T}/2)}\sum_{n,m=1}^{N}\Gamma_{nm}\left[{\hat{a}}_{n}^{†},\left[{\hat{a}}_{m},\hat{O}\right]\right] \end{array}$$

the images of the superoperator $\hat{L}$ and of its conjugate ${\hat{L}}^{†}$ can be derived as an immediate consequence of the above algebraic identities.

## Appendix B. Phase Space Representation for Green’s Function

In this appendix, we employ Equation (28) to obtain the phase space representation for the kernel of the Green’s function also known as the superoperator that governs the temporal evolution of the Glauber–Sudarshan probability quasidistribution (*P*-function) given by

(A14) $$\begin{array}{cc} \; & \hat{\rho }\left(t\right)=\int d\mu \left(\beta \right)P\left(\beta ,t\right)|\beta \rangle \langle \beta |. \end{array}$$

The Glauber–Sudarshan *P*-function is known to be related to the characteristic function $\chi_{N}$ by the Fourier transformation

(A15) $$\begin{array}{cc} \; & P\left(\alpha ,t\right)=\int d\mu \left(\eta \right)e^{\left[\left(\eta ,\alpha^{*}\right)-\left(\eta^{*},\alpha \right)\right]}\chi_{N}\left(\eta ,t\right). \end{array}$$

On the other hand, the relation

(A16) $$\begin{array}{cc} \; & \chi_{N}^{\left(\mathrm{ini}\right)}\left(\alpha \right)=\int d\mu \left(\beta \right)e^{\left[\left(\alpha^{*},\beta \right)-\left(\alpha ,\beta^{*}\right)\right]}P\left(\beta ,0\right) \end{array}$$

gives the characteristic function of the initial state $\chi_{N}^{\left(\mathrm{ini}\right)}$ expressed in terms of P(β,0).

The Green’s function is defined as follows

(A17) $$\begin{array}{c} P(\alpha ,t)=\int d\mu (\beta )G(\alpha ,\beta ,t)P(\beta ,0), \end{array}$$

where G(α,β,t) is the kernel of the superoperator.

We can now substitute Formula (28) into Equation (A15) and use the relation (A16) to derive the Fourier transform of the kernel, $\tilde{G}\left(\eta ,\beta ,t\right)$, which is given by

(A18) $$\begin{array}{cc} \; & \tilde{G}\left(\eta ,\beta ,t\right)=\exp\{n_{T}{\left(|A\left(t\right)\eta \right|}^{2}-{\left|\eta \right|}^{2})\\ \; & +\left(A^{*}\left(t\right)\eta^{*},\beta \right)-\left(A\left(t\right)\eta ,\beta^{*}\right)\}, \end{array}$$

where ${\left|\eta \right|}^{2}\equiv \left(\eta^{*},\eta \right)=\sum_{n=1}^{N}{\left|\eta_{n}\right|}^{2}$. After performing Gaussian integral

(A19) $$\begin{array}{cc} \; & G\left(\alpha ,\beta ,t\right)=\int d\mu \left(\eta \right)e^{\left[\left(\eta ,\alpha^{*}\right)-\left(\eta^{*},\alpha \right)\right]}\tilde{G}\left(\eta ,\beta ,t\right), \end{array}$$

we arrive at the final result for the Green’s function:

(A20) $$\begin{array}{cc} \; & G\left(\alpha ,\beta ,t\right)=\frac{1}{\det B(t)}e^{-(q^{*}\left(t\right),B^{-1}\left(t\right)q\left(t\right))}, \end{array}$$

that has the form of the Gaussian distribution for the vector

(A21) $$\begin{array}{c} q\left(t\right)=A^{†}\left(t\right)\beta -\alpha \end{array}$$

with the covariance matrix given by Equation (29). Note that the expression for the characteristic function (30) leads to similar results for the Wigner function.
